# Cardiovascular Outcomes and Hyperkalemia Risk in Patients With Diabetes, Chronic Kidney Disease and Heart Failure: A Real-World Comparison of Non-steroidal versus Steroidal Mineralocorticoid Receptor Antagonists

**DOI:** 10.7759/cureus.97588

**Published:** 2025-11-23

**Authors:** Ayoyimika O Okunlola, Olayinka Kolawole, Emmanuel Otabor, Abdulraheem Hassan, Michael Hamilton, Laith Alomari, Justin Lam, Abiodun B Idowu

**Affiliations:** 1 Internal Medicine, United Lincolnshire Hospitals NHS Trust, Lincolnshire, GBR; 2 Internal Medicine, Hull University Teaching Hospitals NHS Trust, Hull, GBR; 3 Internal Medicine, Jefferson Einstein Philadelphia Hospital, Philadelphia, USA; 4 Internal Medicine, Saint Peter's University Hospital Rutgers - Robert Wood Johnson Medical School, New Jersey, USA

**Keywords:** chronic kidney disease (ckd), drug-induced hyperkalemia, heart failure, mineralocorticoid receptor antagonists, non-steroidal mineralocorticoid receptor antagonist

## Abstract

Background: Steroidal mineralocorticoid receptor antagonists (MRAs) reduce morbidity in heart failure (HF) but frequently cause hyperkalemia, limiting long-term use. Non-steroidal MRAs such as finerenone offer improved receptor selectivity, but real-world comparative data remain scarce.

Methods: We used the TriNetX Global Collaborative Network to identify adults with type 2 diabetes mellitus (T2DM), chronic kidney disease (CKD), and HF initiated on a non-steroidal (finerenone) or steroidal MRA (spironolactone, eplerenone) between January 2020 and December 2024. Propensity score matching (1:1) yielded 780 patients per cohort. Outcomes included hyperkalemia, arrhythmia, stroke, myocardial infarction (MI), and all-cause mortality.

Results: Non-steroidal MRAs were associated with significantly lower risks of hyperkalemia >5.5 mmol/L (16.0% vs 21.8%; HR 0.683, 95% CI 0.542-0.861; p=0.001) and severe hyperkalemia >6.0 mmol/L (7.6% vs 10.4%; HR 0.690, 95% CI 0.493-0.965; p=0.029). They were also linked to a lower arrhythmia incidence (31.2% vs 44.7%; HR 0.596, 95% CI 0.506-0.703; p<0.001), stroke (6.2% vs 9.9%; HR 0.606, 95% CI 0.422-0.869; p=0.006), and all-cause mortality (6.3% vs 16.5%; HR 0.359, 95% CI 0.258-0.499; p<0.001). MI was numerically lower (10.9% vs 13.5%; HR 0.762, 95% CI 0.572-1.014; p=0.061) but not statistically significant.

Conclusion: In patients with T2DM, CKD, and HF, non-steroidal MRAs were associated with improved safety and cardiovascular outcomes compared with steroidal MRAs. However, the observational study design and, hence, the limitation in data that can be collected warrant cautious interpretation. Prospective randomized head-to-head trials are warranted before adaptation in clinical practice.

## Introduction

Mineralocorticoid receptor antagonists (MRAs) represent an established therapeutic class in the management of patients with heart failure (HF) and chronic kidney disease (CKD). Their cardioprotective benefits are well documented in randomized controlled trials, including the RALES and EPHESUS studies, which demonstrated significant reductions in morbidity and mortality with spironolactone and eplerenone [1,2]. However, despite these benefits, steroidal MRAs are associated with frequent hyperkalemia, particularly in individuals with type 2 diabetes mellitus (T2DM) and CKD, where impaired renal potassium excretion heightens the risk [3]. This safety concern often results in dose reduction or discontinuation, limiting the long-term effectiveness of therapy and contributing to underutilization in routine practice [4].

In recent years, non-steroidal MRAs such as finerenone and esaxerenone have emerged as alternatives with distinct pharmacological properties. Unlike steroidal MRAs, which exhibit broad receptor activity, non-steroidal MRAs bind selectively to the mineralocorticoid receptor with less interaction at androgen and progesterone receptors [5]. This improved selectivity may translate into reduced off-target effects and potentially lower the risk of hyperkalemia [6]. Furthermore, preclinical studies suggest that non-steroidal MRAs exert stronger anti-inflammatory and anti-fibrotic effects on cardiac and renal tissues compared with spironolactone [7].

The clinical efficacy of non-steroidal MRAs has been evaluated in landmark randomized trials. The FIDELIO-DKD trial established that finerenone significantly reduced the risk of CKD progression and cardiovascular events in patients with T2DM and CKD [8]. Similarly, the FIGARO-DKD trial demonstrated reductions in major adverse cardiovascular events, particularly HF hospitalization [9]. A pooled analysis of these two trials, the FIDELITY dataset, confirmed consistent cardiovascular and renal benefits across a broad range of patients with T2DM and CKD [10]. Importantly, while hyperkalemia events were observed, they were generally manageable and occurred less frequently than anticipated from prior experiences with spironolactone.

Despite these promising results, a key gap remains: the pivotal trials compared non-steroidal MRAs against placebo, not against active treatment with steroidal MRAs. As such, it remains uncertain whether non-steroidal MRAs offer a tangible advantage over steroidal agents in real-world settings, where polypharmacy, comorbidities, and adherence issues are prevalent. Observational data directly comparing these drug classes are limited, and robust evidence is needed to inform guideline recommendations.

This study therefore leverages the TriNetX Global Collaborative Network to evaluate cardiovascular outcomes and hyperkalemia risk in patients with T2DM, CKD, and HF treated with non-steroidal versus steroidal MRAs. By applying propensity score matching (PSM) to balance demographics, comorbidities, and background therapies, this analysis aims to provide real-world evidence on the comparative safety and effectiveness of these therapeutic classes.

## Materials and methods

Data source

We conducted this retrospective cohort study using the TriNetX Global Collaborative Network, a federated health research platform that aggregates anonymized electronic health record (EHR) data from more than 148 healthcare organizations across North America, Europe, and Asia [11]. The network includes academic medical centers, integrated delivery networks, and specialty hospitals, providing access to over 100 million patient records. Data are updated in near real time and include demographics, diagnoses, laboratory values, procedures, medications, and mortality.

Study population and exposure groups

We identified adult patients with concurrent diagnoses of type 2 diabetes mellitus (T2DM; ICD-10 E11), chronic kidney disease (CKD; N18), and heart failure (HF; I50.2, I50.3). Eligible individuals were those whose treatment was initiated between January 1, 2020, and December 31, 2024, with either a non-steroidal MRA (finerenone) or a steroidal MRA (spironolactone or eplerenone). The index date was defined as the first prescription for the respective MRA during this period.

Two mutually exclusive cohorts were defined for analysis. The first consisted of patients who initiated treatment with a non-steroidal MRA, and the second included those who started therapy with a steroidal MRA. Patients were not permitted to contribute to both groups, ensuring distinct exposure categories. To maintain a new-user design, individuals with prescriptions for an agent from the opposite MRA class in the six months preceding the index date were excluded.

Covariates and matching

To reduce confounding, baseline covariates were assessed during the six months preceding the index date, including demographics, comorbidities (hypertension, obesity, tobacco use, prior cardiovascular disease), medications (SGLT2 inhibitors, ACE inhibitors/ARBs, beta-blockers, statins, aspirin, diuretics), and laboratory values (eGFR, HbA1c).

Propensity scores were estimated via logistic regression using these covariates, followed by 1:1 greedy nearest-neighbor matching without replacement and a caliper of 0.10 standard deviations of the propensity-score distribution. Covariate balance was evaluated using standardized mean differences (SMD), with < 0.10 indicating adequate balance.

Study cohort and follow-up

Before matching, 109,375 patients met inclusion criteria, including 785 in the non-steroidal MRA group and 108,590 in the steroidal MRA group. After 1:1 PSM, 1,560 patients remained (780 per group). All patients were followed for 12 months from the index date for outcome assessment.

Outcomes

The primary safety outcomes were hyperkalemia (serum potassium > 5.5 mmol/L) and severe hyperkalemia (> 6.0 mmol/L). Secondary efficacy outcomes included arrhythmia (defined as a composite of atrial fibrillation/flutter, ventricular tachycardia, or ventricular fibrillation), myocardial infarction (MI), stroke (ischemic stroke or cerebral infarction), and all-cause mortality.

Statistical analysis

Baseline characteristics were summarized using descriptive statistics. After PSM, survival analyses were performed using Kaplan-Meier estimates with log-rank tests. Cox proportional-hazards models were used to estimate hazard ratios (HRs) with 95% confidence intervals (CIs) for each outcome. All analyses were performed within the TriNetX platform, which applies validated methods for cohort creation, matching, and outcome assessment [11].

Ethical considerations

All data were de-identified in accordance with the Health Insurance Portability and Accountability Act (HIPAA) and the General Data Protection Regulation (GDPR). As the analysis used anonymized records from the TriNetX platform, the study qualified for exemption from institutional review board (IRB) approval and informed consent requirements.

## Results

Baseline characteristics before and after PSM are presented in Table 1. Matching achieved good balance across most covariates, with the majority of standardized mean differences below the 0.10 threshold. While 'White race' (SMD 0.116) and 'Chronic kidney disease' (SMD 0.106) were slightly above this target, this was considered an acceptable balance. After PSM, both cohorts comprised 780 patients. Baseline demographics, comorbidities, and medication use were well-balanced. Mean age was 72 years, 36-38% were female, approximately 16% were Black, and mean HbA1c was approximately 7.3%.

**Table 1:** Baseline Characteristics Before and After Propensity Score Matching Values are presented as mean ± standard deviation (SD) for continuous variables and number (percentage) for categorical variables. SMD: Standardized mean difference; eGFR: estimated glomerular filtration rate; MRA: mineralocorticoid receptor antagonist; PSM: propensity score matching

Characteristic	Non-steroidal MRA (Pre-PSM, n = 785)	Steroidal MRA (Pre-PSM, n = 108 590)	Non-steroidal MRA (Post-PSM, n = 780)	Steroidal MRA (Post-PSM, n = 780)	SMD (Post-PSM)
Demographics					
Age, mean ± SD	71.9 ± 10.6	72.1 ± 12.0	72.0 ± 10.6	71.9 ± 12.1	0.002
White race	392 (49.9%)	66, 413 (61.2%)	392 (50.3%)	437 (56.0%)	0.116
Black	124 (15.8%)	24, 256 (22.3%)	124 (15.9%)	118 (15.1%)	0.021
Asian race	177 (22.5%)	3, 614 (3.3%)	172 (22.1%)	146 (18.7%)	0.083
Unknown ethnicity	136 (17.3%)	27, 011 (24.9%)	136 (17.4%)	134 (17.2%)	0.007
Female sex	296 (37.7%)	45, 073 (41.5%)	296 (37.9%)	274 (35.1%)	0.059
Male sex	478 (60.9%)	59, 924 (55.2%)	473 (60.6%)	495 (63.5%)	0.058
Comorbidities					
Tobacco use	60 (7.6%)	10, 495 (9.7%)	60 (7.7%)	69 (8.8%)	0.042
Chronic kidney disease	762 (97.1%)	80, 311 (74.0%)	757 (97.1%)	769 (98.6%)	0.106
Obesity	360 (45.9%)	49 556 (45.6%)	359 (46.0%)	362 (46.4%)	0.008
Hypertension	714 (91.0%)	93, 700 (86.3%)	710 (91.0%)	723 (92.7%)	0.061
Medications					
β-blocking agents	685 (87.3%)	98, 577 (90.8%)	681 (87.3%)	680 (87.2%)	0.004
SGLT2 inhibitors	471 (60.0%)	33, 367 (30.7%)	466 (59.7%)	477 (61.2%)	0.029
Aspirin	583 (74.3%)	83, 340 (76.7%)	580 (74.4%)	585 (75.0%)	0.015
ACE inhibitors/ARBs	503 (64.1%)	62, 088 (57.2%)	498 (63.8%)	494 (63.3%)	0.011
Statins	683 (87.0%)	90, 792 (83.6%)	678 (86.9%)	679 (87.1%)	0.004
Diuretics	699 (89.0%)	102, 978 (94.8%)	695 (89.1%)	692 (88.7%)	0.012
Laboratory					
eGFR 30–60mL/min/1.73 m²	683 (87.0%)	91,789 (84.5%)	678 (86.9%)	686 (87.9%)	0.031
HbA1c (%), mean ± SD	7.4 ± 1.5	7.1 ± 1.8	7.4 ± 1.5	7.3 ± 1.7	0.048

In the matched cohort (n=780), initiation of non-steroidal MRAs was associated with a meaningful decrease in hyperkalemia cases, as detailed in Table 2. After one year, 16.0% of patients on these agents had potassium levels exceeding 5.5 mmol/L, compared to 21.8% of those on steroidal MRAs (HR 0.683, 95% CI 0.542-0.861, p=0.001). Severe hyperkalemia (>6.0 mmol/L) was also less common, occurring in 7.6% versus 10.4% of patients, respectively (HR 0.690, 95% CI 0.493-0.965, p=0.029).

**Table 2:** Comparison of Clinical Outcomes Between Non-steroidal and Steroidal MRAs at 12 Months Hazard ratios (HRs) with 95% confidence intervals (CIs) were estimated using Cox proportional-hazards models. p-values were assessed using the log-rank test. MRA: Mineralocorticoid receptor antagonist

Outcome	Non-steroidal MRA (n=780)	Steroidal MRA (n=780)	Hazard Ratio (95% CI)	p-value
Hyperkalemia >5.5 mmol/L, n (%)	125 (16.0%)	170 (21.8%)	0.683 (0.542–0.861)	0.001
Hyperkalemia >6.0 mmol/L, n (%)	59 (7.6%)	81 (10.4%)	0.690 (0.493–0.965)	0.029
Arrhythmia, n (%)	243 (31.2%)	349 (44.7%)	0.596 (0.506–0.703)	<0.001
Stroke, n (%)	48 (6.2%)	77 (9.9%)	0.606 (0.422–0.869)	0.006
Myocardial infarction, n (%)	85 (10.9%)	105 (13.5%)	0.762 (0.572–1.014)	0.061
All-cause mortality, n (%)	49 (6.3%)	129 (16.5%)	0.359 (0.258–0.499)	<0.001

The use of steroidal MRAs was also linked to a substantially higher frequency of arrhythmias. These events affected 44.7% of steroidal MRA users, in contrast to 31.2% of those on non-steroidal agents (HR 0.596, 95% CI 0.506-0.703, p<0.001). The absolute risk difference of 13.5% suggests clinically meaningful prevention of rhythm disturbances.

Furthermore, stroke rates were lower with non-steroidal MRAs, occurring in 6.2% of patients compared to 9.9% with steroidal MRAs (HR 0.606, 95% CI 0.422-0.869, p=0.006), equating to a 39% relative risk reduction. While MI rates were numerically lower with non-steroidal MRAs (10.9% vs 13.5%), this difference was not statistically significant (HR 0.762, 95% CI 0.572-1.014, p=0.061), though it suggests a potential cardiovascular benefit.

Most strikingly, mortality was markedly lower in the non-steroidal MRA group. At 12 months, the death rate was 6.3%, compared to 16.5% for steroidal MRA patients (HR 0.359, 95% CI 0.258-0.499, p<0.001). This represents a 64% relative reduction in death, with survival advantages appearing early and increasing over time.

## Discussion

In this large, real-world propensity score-matched analysis, we found that non-steroidal MRAs were associated with significantly improved safety and efficacy compared with steroidal MRAs among high-risk patients with T2DM, CKD, and HF. Our principal finding is that non-steroidal MRA use was linked to lower rates of both moderate and severe hyperkalemia. Furthermore, we observed significant reductions in arrhythmia, stroke, and all-cause mortality, though the trend toward lower MI did not reach statistical significance. These results complement existing trial data by providing head-to-head comparative evidence in a routine clinical practice setting.

The results align with and extend findings from prior clinical trials. The RALES and EPHESUS trials established spironolactone and eplerenone as effective steroidal MRAs, showing significant reductions in morbidity and mortality in HF populations [1,2]. However, registry analyses have repeatedly demonstrated high rates of hyperkalemia and discontinuation in real-world practice [3].

By contrast, the FIDELIO-DKD and FIGARO-DKD trials of finerenone demonstrated reductions in CKD progression and cardiovascular events in patients with T2DM and CKD [8,9]. A pooled analysis of these trials (FIDELITY) confirmed consistent benefits across a wide spectrum of patients [10]. Importantly, although hyperkalemia occurred more frequently with finerenone than placebo, the incidence was lower than historical rates observed with spironolactone.

Our study differs by directly comparing non-steroidal and steroidal MRAs in routine care. The observed reduction in hyperkalemia and cardiovascular outcomes with non-steroidal MRAs provides supportive real-world evidence that these agents may offer advantages over steroidal MRAs, beyond what could be inferred from placebo-controlled trials.

The observed benefits may be attributable to distinct pharmacological properties of non-steroidal MRAs. Unlike spironolactone and eplerenone, which have relatively broad receptor activity, finerenone exhibits high selectivity for the mineralocorticoid receptor without significant affinity for androgen or progesterone receptors [5]. This specificity is thought to reduce off-target effects and may contribute to improved tolerability.

Moreover, finerenone demonstrates unique tissue distribution, with relatively greater activity in the myocardium and vasculature compared with the kidney [6,7]. Preclinical studies suggest that this may lead to stronger anti-fibrotic and anti-inflammatory effects, which may underlie the observed reductions in arrhythmias and stroke [9]. The reduction in mortality seen in our analysis could reflect not only lower hyperkalemia rates, but also attenuation of progressive myocardial and vascular remodeling.

Strengths and limitations

Strengths of our study include the use of a large, multinational dataset and robust PSM, which minimized confounding by balancing demographics, comorbidities, and concomitant therapies. The inclusion of both laboratory and clinical outcomes enhances the reliability of findings.

However, limitations must be acknowledged. First, as an observational analysis, residual confounding cannot be excluded despite careful matching. Variables such as left ventricular ejection fraction, New York Heart Association class, and adherence were not available. Second, the follow-up duration was limited to 12 months; longer-term outcomes may differ. Third, outcome ascertainment relied on ICD coding and electronic health records, which may underestimate true event rates. Finally, the striking mortality benefit observed, while biologically plausible, was larger than anticipated from trial data and must be interpreted with caution.

Clinical implications

The findings have important implications for clinical practice. Patients with T2DM, CKD, and HF represent a particularly vulnerable group, in whom both hyperkalemia and cardiovascular events are common. By reducing the risk of hyperkalemia, non-steroidal MRAs may allow patients to remain on guideline-directed therapies longer, thereby maximizing long-term benefits. The observed reduction in arrhythmias and stroke also highlights a potential role for these agents in reducing thromboembolic risk and sudden cardiac death.

Importantly, our findings align with emerging guideline trends. The 2022 American Diabetes Association (ADA) Standards of Care and European Society of Cardiology (ESC) heart failure guidelines already recommend finerenone in patients with T2DM and CKD at risk of cardiovascular events [12,13]. However, explicit comparisons with steroidal MRAs are not yet addressed. Our results provide real-world evidence to support preferential use of non-steroidal MRAs in high-risk patients where both classes are available.

The observational nature of the study implies that certain parameters such as NYHA class of heart failure, the exact value of the ejection fraction, and adherence to medication were unaccounted for and the magnitude of the observed mortality benefit therefore warrants cautious interpretation when applying these findings to clinical practice. Prospective, head-to-head randomized trials are necessary to validate these results and inform future clinical guidelines.

Future directions

Future research should focus on head-to-head randomized controlled trials comparing steroidal and non-steroidal MRAs in diverse HF populations, including those with preserved ejection fraction. Longer-term follow-up studies are needed to confirm survival benefits and assess renal endpoints, such as progression to end-stage kidney disease. In addition, subgroup analyses by race, sex, and concomitant use of SGLT2 inhibitors or GLP-1 receptor agonists would provide important insights into differential responses.

## Conclusions

In this large, real-world analysis of high-risk patients with type 2 diabetes, CKD, and HF, non-steroidal MRAs were associated with superior safety and cardiovascular outcomes compared to steroidal MRAs. Specifically, their use was associated with significantly lower risks of hyperkalemia, arrhythmia, stroke, and a substantial reduction in all-cause mortality. These findings position non-steroidal MRAs as a potentially preferable therapeutic option, offering improved tolerability in this vulnerable population. The observational design of the study naturally limits the depth and type of data that can be captured, meaning the findings particularly the magnitude of the mortality benefit should be interpreted with caution. To strengthen the evidence base, well-designed prospective, head-to-head randomized trials are needed to confirm these results and guide future clinical recommendations.
